# Effect of 3D-Printed Honeycomb Core on Compressive Property of Hybrid Energy Absorbers: Experimental Testing and Optimization Analysis

**DOI:** 10.3390/ma17020522

**Published:** 2024-01-22

**Authors:** Rita de Cássia Silva, Gabriel Martins de Castro, Alessandro Borges de Sousa Oliveira, Augusto César de Mendonça Brasil

**Affiliations:** 1 Department of Automotive Engineering, Group of Modeling and Simulation of Vehicle Systems, Gama College, University of Brasilia (UnB), Brasilia 72444-240, Brazil; abso@unb.br; 2 Post-Graduation in Transport, Group of Modeling and Simulation of Vehicle Systems, Campus Darcy Ribeiro, University of Brasilia (UnB), Brasilia 70910-900, Brazil; castro.mgabriel@outlook.com (G.M.d.C.); ambrasil@unb.br (A.C.d.M.B.)

**Keywords:** hybrid energy absorbers, honeycomb, PET-G polymer, ABS polymer, optimization method

## Abstract

This paper presents an innovative method of constructing energy absorbers, whose primary function is to effectively transform kinetic energy into strain energy in events with high deformation rates. Hybrid specimens are proposed considering thin-walled windowed metallic tubes filled with 3D-printed hexagonal honeycombs made of PET-G and ABS thermoplastic. The patterned windows dimensions vary from 20 × 20, 20 × 30, 15 × 20 and 15 × 30 mm^2^. Although using polymers in engineering and thin-walled sections is not new, their combination has not been explored in this type of structure designed to withstand impacts. Specimens resist out-of-plane quasi-static axial loading, and test results are analyzed, demonstrating that polymer core gives the samples better performance parameters than unfilled samples regarding energy absorption (*E_a_*), load rate (*LR*), and structural effectiveness (*η*). An optimization procedure using specialized software was applied to evaluate experimental results, which led to identifying the optimal window geometry (16.4 × 20 mm^2^, in case) and polymer to be used (ABS). The optimized sample was constructed and tested for axial compression to validate the optimization outcomes. The results reveal that the optimal sample performed similarly to the estimated parameters, making this geometry the best choice under the test conditions.

## 1. Introduction

Polymers and their nanocomposites have become essential in engineering and technology due to some advantages imposed by such materials. Some benefits of using these materials are resistance to corrosion, lightweight, strength, stiffness, and fatigue resistance [1]. Numerous engineering applications related to polymers are present in the automotive industry nowadays, as emphasized by the work of [2]. The authors predicted this trend would continue, which has proven to be the case, as shown in the following text.

Honeycomb structures are widely used in various industries, such as aerospace, automotive, construction, and packaging, due to their exceptional mechanical properties. Concerning the material selection, such a choice depends on the specific application requirements. Common materials are aluminum, steel, titanium, and composite materials. An analysis of the latest trends considering hybrid components of lightweight materials aimed at structural use was carried out by Rubio et al. [3]. They concluded that from the 142 scientific publications consulted for the work, 41% considered the hybrid components metal-polymer reinforced with fibers and 28% metal-polymer, i.e., almost 71% of the consulted articles consider such a combination, reinforcing the interest in the subject.

Thermoplastic materials, such as PET-G (polyethylene terephthalate glycol), ABS (acrylonitrile butadiene styrene), and PLA (polylactic acid) combined with other materials, among them, metallic materials [4,5] have experienced increased use recent years. The present research proposes a potential application for hybrid energy absorbers in vehicle safety. These absorbers consist of hollow metallic tubes filled with a polymer honeycomb core. The subject of energy absorbers focused on vehicle safety has aroused so much interest in the scientific community that [5] presented an extensive literature survey centered on these structures. The authors comprehensively summarized different research approaches and aspects related to such structures. 

Multiple techniques are available to manufacture cellular core structures, such as adhesive bonding, resistance welding, brazing, diffusion bonding, and thermal fusion. However, the most common manufacturing method is adhesive bonding [6]. The expansion and corrugation methods are the basis of the adhesive bonding manufacturing method. Nowadays, Fused Deposition Modelling, FDM^®^, is a technique used for three-dimensional (3D) printing structures using polymer fiber or fiber-reinforced composites (FRC), and it is currently one of the most rapidly expanding additive manufacturing techniques. Progress in 3D printing using the FDM^®^ technique can be seen by examining numerous scientific works. Wickramasinghe et al. [7] discussed ways to characterize and classify defects found in 3D printing using polymers and composites, applying the technique of FDM^®^ in their work. Daminabo et al. [8] presented in their work a review that provides an understanding of current additive manufacturing (AM) techniques, particularly emphasizing extrusion-based technologies like fused deposition modeling (FDM^®^) and direct ink writing. These techniques are preferred due to their scalability, cost-effectiveness, and the ability to process a diverse range of materials, as emphasized by the authors. Sharma and Rai [9] discussed the growing trend of additive manufacturing in the modern industrial market, focusing on the fused-based modeling technique. The review explores some of the following aspects of applications and materials used in fused-based modeling. As a result, this text suggests that additive manufacturing is an upcoming trend in the industry.

PET-G and ABS polymer fibers were utilized in the present study to manufacture honeycomb cores through 3D printing with the FDM^®^ technique. The authors had previously conducted experimental tests on both polymers according to ASTM standards C365 [10] and D638 [11]. These standards determine the compressive strength and modulus of sandwich cores under quasi-static compressive loads, as well as the tensile properties, respectively. The findings were shown in [12] and, allowing for improvements in the 3D printing process, resulted in optimized configurations to produce honeycomb parts. The honeycomb structures were subjected to out-of-plane compression loading. Similar scientific literature with an investigative focus on mechanical properties is available in [13], which investigated the mechanical properties of 3D-printed honeycomb structures made of PET-G polymer under in-plane compression. The experimental testing took into account different infill density values and printing orientations, affecting the samples’ energy absorption. Wang et al. [14] evaluated the capacity of printed polymeric honeycombs packed with foamed concrete to endure in-plane crushing. Recently, Durga Rajesh et al. [15] conducted a study on the mechanical properties of ABS, PET-G, and PLA specimens produced through 3D printing. They aimed to create a guideline for thermoplastic fabrication and provide essential information on the mechanical aspects of functional thermoplastics fabricated using the FDM^®^ technique. Thus, scientific research has confirmed that understanding the mechanical properties of these materials is crucial due to their broad range of applications in diverse fields of knowledge.

Bates et al. [16] discussed the favorable mechanical properties of fused filament in thermoplastic polyurethanes (TPUs) concerning its capability to manufacture flexible honeycomb structures aiming to optimize energy absorption applications. The authors studied the effect of how grading methodologies could influence the energy absorption and damping behavior of these structures using such a material. They used the 3D printing procedure and quasi-static testing. Tao et al. [17] used square hierarchical honeycombs made of VeroWhitePlus, an acrylic polymer, through 3D printing, with the goal of investigating the samples’ in-plane mechanical properties and energy absorption. Analytical equations for Young’s modulus and the compressive strength of those samples were developed. Rahman and Koohbor [18] also applied the technique of density gradation to enhance the load-bearing and energy absorption efficiency of polymeric cellular structures using polyurethane. The optimization proposed was based on virtual experiments. Tan et al. [19] studied graded re-entrant hierarchical honeycomb sandwich panels, focusing on their crashworthiness performance under in-plane compression. In the study, energy absorption capability was evaluated, as well as the effect of the gradient, arranged orientation of the panel core, and impact velocities. Cheng et al. [20] reported the failure and recovery mechanisms of 3D-printed composite honeycomb composites (PLA honeycomb reinforced by continuous carbon fiber). In the study, the authors highlighted the enhancement of composite mechanical properties and their ability to recover shape under heat excitation. Menegozzo et al. [21] presented a new design of honeycomb cell geometry aiming to overcome the limitations of hexagonal honeycombs. The design deals with low stiffness and energy absorption under loaded with significant lateral components. Specimens are 3D-printed, made from ABS thermoplastic material, and quasi-static compression loading. Acanfora et al. [22] presented an innovative shock absorber using additive manufacturing technology. The advanced sandwich shock absorber combined thermoplastic (polypropylene) and fiber-reinforced thermoset composites (carbon fiber reinforced polymers—CFRP) to achieve optimal mechanical efficiency while minimizing mass and volume. The initial design featured a polypropylene honeycomb core and CFRP composite external skins, which demonstrated superior crashworthiness performance overall. Bochnia et al. [23] presented a study concerning the rheological and mechanical properties of a resin (MED610) broadly used in medicine. They test various sample types, and one of them is hexagonal cellular structure. The focus was to verify the influence of orientation on the working platform of the 3D printer in mechanical properties. In addition, the research considers both cellular and thin-walled models.

According to the research above, the following findings may be highlighted: (a) numerous applications can be recognized in automotive engineering, using just polymers or hybrid structures; (b) various techniques can be employed to manufacture honeycomb structures, but it can be identified from the researches that the FDM^®^ technique has experienced significant progress in engineering applications and (c) the studies focused on understanding material mechanical properties and the structural arrangement of distribution and cell shape, considering: loading orientation, polymeric material type, and cell graded density. The primary aim was to determine the energy absorption capacity and crashworthiness performance.

The method of producing polymeric honeycomb (ABS and PET-G) using 3D printing techniques is not new, as exemplified by the studies in this discussion. However, using it as a core material for square steel thin-walled energy absorbers presents a novel approach to engineering solutions. Also, the metallic tubes are windowed at half height, considering openings of 20 × 20 mm^2^, 20 × 30 mm^2^, 15 × 30 mm^2^, and 15 × 20 mm^2^. Such openings, in addition to the polymer core, positively affected crashworthiness by increasing energy absorption capacity, improving load ratio (LR), and enhancing structural effectiveness (*η*). The hybrid tubes were under quasi-static axial compression loading. An optimization study was carried out based on the performance parameter results of all specimens, which allowed for identifying the best window dimension and core material. This optimized sample was built and tested under quasi-static compression, and the results obtained for the performance parameters were what was foreseen in the optimization study. Such behavior highlights the efficiency of the applied method.

## 2. Energy Absorber Performance Parameters: Short Description

The performance parameters used to evaluate the crashworthiness ability of energy absorbers are well-defined in scientific literature, as shown [24,25,26,27]. Consequently, the authors summarized these parameters in Table 1, indicating some research issues in the second column. Equations (1)–(6) will be used to calculate the specimens’ parameters under axial quasi-static compression, Section 4.

## 3. Materials and Methods

### 3.1. Hybrid Energy Absorber Geometry Design and Applied Material

The novel hybrid energy absorber proposed in the present research was constructed considering the following requirements: (1) the ability to increase the energy absorption of conventional thin-walled metallic tubes (unfilled); (2) the core presence in thin-walled energy absorbers guarantees an increase in the energy absorption, as cited by [24,28,29], but the purpose of thin-walled metallic tubes filled with a 3D-printed hexagonal honeycomb of polymeric thermoplastic material may be an upcoming trend due to the possibility of scale production, low-cost, and the capacity to process a diverse range of geometry using different materials, (3) patterned windows can change the buckling mode of the specimens considering the ratio width/height, as pointed out by [30]. Windowed specimens can influence the peak force magnitude, reducing it due to the escape area as it represents when dynamically loading. Regarding vehicle safety, this would reduce the risk of neck and head injuries for occupants, and (4) the proposed height of the specimens makes them suitable for insertion in small spaces, such as the side doors of vehicles, where they can act as side protection and replace side protection bars in various vehicle models.

**Figure 1:**
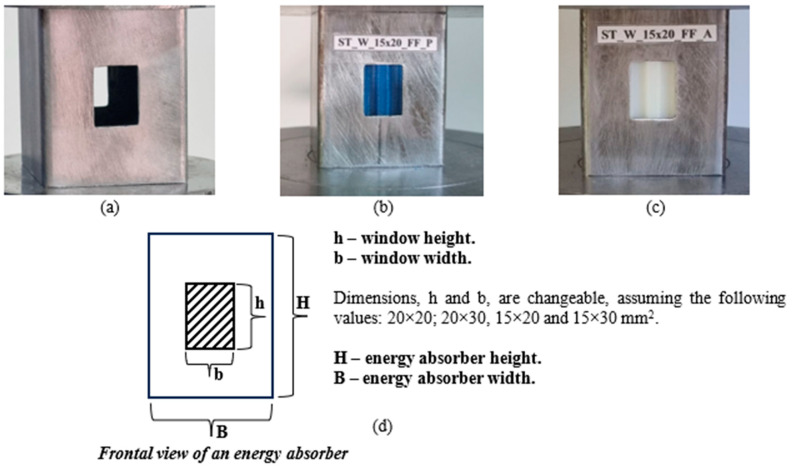
Design illustration of the thin-walled hybrid energy absorber with window (**a**) unfilled sample with dimension window of 15 × 20 mm^2^ (b/h—b = 15 mm and h = 20 mm) (**b**) filled sample with PET-G (**c**) filled sample with ABS (**d**) schematic of the energy absorber considering the front view.

The novel hybrid energy absorber was designed to meet the earlier requirements, considering thin-walled windowed metallic tubes filled with 3D-printed hexagonal honeycombs made of PET-G and ABS thermoformable thermoplastic. The patterned window dimensions vary from 20 × 20, 20 × 30, 15 × 20, and 15 × 30 mm^2^, as shown in Figure 1a–d. These openings were placed at the center of opposite faces, as shown in Figure 1a,d. The major ratio—width/height = b/h—was 1.0 (one), which corresponded to the sample 20 × 20 mm^2^, followed by the ratios 0.75 (15 × 20 mm^2^), 0.67 (20 × 30 mm^2^) and 0.5 (15 × 30). The samples’ height was 60 mm (H) and 50 mm in width (B), Figure 1d, featuring a square thin-walled cross-section with 1.55 mm thickness. The thin-walled tubes were purchased from commercial suppliers, and all specimens were fabricated from the same material lot.

The specimens were sliced and machined to obtain windowed samples with specific patterns. Song et al. [30] designed thin-walled windowed metallic tubes and submitted them to quasi-static axial compression. 

Their findings have shown that such a design could be grouped into three main collapse modes: symmetric, extensional, and diamond, depending on the window dimensions and position relative to the tube face. Furthermore, their study indicates that the window’s width is relatively narrow when set to symmetric mode. By increasing the window’s height, the collapse mode becomes more irregular. 

In the present work, we adopted the methodology presented in Figure 11 in the research conducted by [30] to establish that all unfilled samples would primarily fail symmetrically. Selecting the ratios a/c and b/H to infer the failure modes is essential. These ratios are required inputs for utilizing the graphic mentioned. The dimensions for the windows are represented by variables ‘*a*’ and ‘*b*’ for width and height, respectively. “*c*” and ‘*H*’ represent the cross-section dimension and mean height between window layers, as defined in the study by [30]. For the current study, the values of ‘*H*’ and ‘*c*’ are 60 mm (because there was only one layer in the energy absorber) and 50 mm, respectively. The variables ‘*a*’ and ‘*b*’ assume the following values, considering the window dimensions, then, for 20 × 20 mm^2^ (*a* and *b* is 20 mm); for 20 × 30 mm^2^ (*a* = 20 mm and *b* = 30 mm); 15 × 20 mm^2^ (*a* = 15 mm and *b* = 20 mm) and 15 × 30 mm^2^ (*a* = 15 mm and *b* = 30 mm).

In the present study, the authors labeled the specimens as ST_W_DD_FF_MM, which stands for:

- ST (steel)—the tube material;
- W (window)—the presence of a window followed by its dimensions (DD), for example, W_15 × 20 mm^2^;
- FF (filled)—the sample has a PET-G or ABS core;
- MM (material)—the type of honeycomb material used, which could be ‘P’ for PET-G in Figure 1b or ‘A’ for ABS in Figure 1c.

This naming convention will be consistently used in this paper, particularly in Section 4. The metal tube material was SAE 1010, verified in tension according to the standard [31], corresponding to the standard specimen denoted ‘sheet-type, 12.5 mm’. Three samples were tested, giving the mean values of Young’s modulus and yield stress of about 195 GPa and 182 MPa, respectively. 

The material used for manufacturing honeycomb cores and tension specimens (Section 3.2) was blue metal PET-G filament and natural ivory for ABS, 1.75 mm in diameter for both, manufactured by 3D Fila, a Brazilian multinational manufacturer based in the city of Belo Horizonte, Brazil. Polyethylene Terephthalate Glycol (PET-G) is a thermoplastic that shares many of the properties of PET [32,33] and is widely used in a broad range of applications [34,35,36,37,38]. Furthermore, due to its outstanding mechanical and thermal properties, this material has become frequently used in 3D printing. Presently, it is ranked as the third most used thermoplastic, with only ABS and PLA being more widespread. Acrylonitrile Butadiene Styrene (ABS) is an extensively used amorphous polymer in engineering applications [39,40,41], which has led to numerous studies on its mechanical, environmental behavior, and thermal properties.

PET-G has a glass transition temperature of about 76.5 °C and a melting temperature of 180.6 °C, while ABS has 101.7 °C and 184.3 °C, respectively. These temperatures were defined by the authors using differential scanning calorimetry (DSC), a technique used to investigate the response of polymers to heating.

The polymer samples were analyzed from 25 °C to 600 °C at a heating rate of 10 °C/min in a simultaneous (TGA-DSC) thermal analyzer (Q600 SDT, TA Instruments, New Castle, DE, USA) under nitrogen atmosphere at a flow rate of 50 mL/min. Samples weighing 10 ± 0.5 mg were deposited on an aluminum pan.

**Figure 2:**
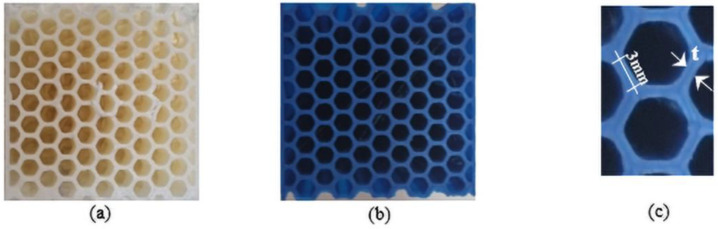
(**a**,**b**) Upper-view of the honeycomb core made of ABS and PET-G, respectively; (**c**) cell dimensions.

The slicer software used to split the 3D models into horizontal layers is Cura 3.0 by UltiMaker (Watermolenweg, The Netherlands). Figure 2 depicts the cores in thermoplastic ABS and PET-G. 

According to [25,42], the mechanical properties of a honeycomb core design are mainly affected by the cell angle, thickness, and length of the cell wall. From [43], these dimensions were adopted as 120°, 0.4 mm, and 3 mm, respectively, and it is possible to determine the honeycomb density (HD) that values 260.7 kg/m^3^. The HD depends on both the cell thickness and length; thus, a smaller cell size leads to a more robust honeycomb core. In future research, we will vary this parameter to reduce the cell number but keep the core behavior as a cellular structure.

**Figure 3:**
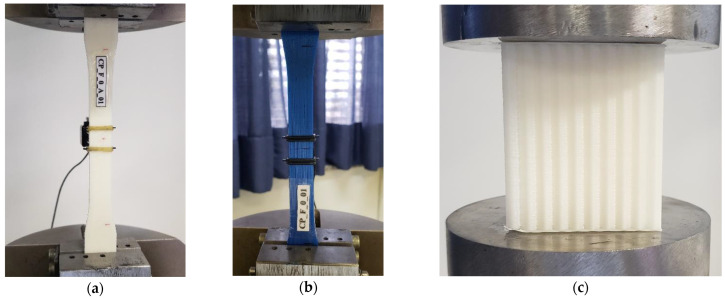
(**a**) ABS tension sample placed in the Universal Machine testing; (**b**) PET-G tension sample placed in the Universal Machine testing; (**c**) ABS compression sample placed in the Universal Machine testing.

Before building the hybrid energy absorbers, compression and tension tests were conducted on 3D-printed pieces of PET-G and ABS polymers used in the core to determine their mechanical properties, such as flow stress, tensile strength at break, modulus of elasticity, elongation, ultimate strength, deflection stress, and compressive modulus. Three specimens were submitted in compression, according to [10], and five in tension (Type I), as subscribed in [11]. The compression specimens had a square cross-section of 2500 mm^2^; for a honeycomb core, the cross-sectional area was defined in the plane of the cells, which is perpendicular to the orientation of the cell’s walls. Figure 3 illustrates the specimens before testing in tension and compression. The methodology was the same for both polymers. The findings of these experiments are outlined in Section 3.2.

### 3.2. Polymer Sample Results under Tension and Compression Tests according to ASTM Standards

The printing parameters were set in accordance with the manufacturer’s recommendations. Thus:

- Printing temperature: 255 °C;
- Platform temperature: 70 °C;
- Printing speed: 55 mm/min, set lower than the manufacturer’s recommendation to improve print quality and prevent interlayer air gaps.

The quality of FDM^®^ parts can be improved by selecting appropriate geometrical parameter settings on the 3D printer. These parameters for the tension samples were building orientation (flat), raster angle (0°), infill density (100%), and layer thickness (0.2 mm), and for compression specimens, the changes concerned the raster angle (90°) and the layer thickness (0.4 mm). Over the years, some authors have studied the influence of these parameters on the mechanical properties of the printed parts [44,45,46]. The raster angle of 0º and the build orientation may maximize the values of stress because both led the fused filament to be put in the pull direction, see [45,47].

The build orientation of a 3D-printed sample can affect its mechanical properties, especially ductility. Ref. [45] state that flat orientation produces the best results. The raster angle describes the direction in which the fused filament deposition is laid in relation to the loading of the part. However, there is no agreement in the literature on what the optimal raster angle should be. In tension tests, a raster angle of 0° indicates that the filament deposition was aligned with the load direction, resulting in improved mechanical strength of the specimen [44]. As stated by [45], the change in infill density mainly determines the printed parts’ tensile strength. Additionally, it was noted that mechanical properties improve as layer thickness increases. The mechanical testing took place in an environment at room temperature.

**Figure 4:**
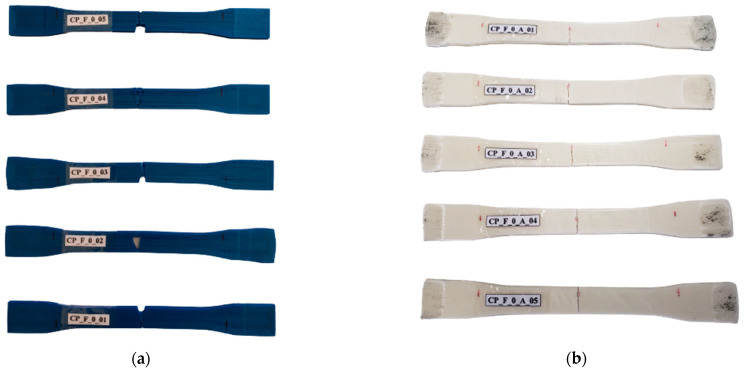
Specimens 3D-printed under flat orientation after tension testing: (**a**) specimens in PET-G and (**b**) specimens in ABS.

To prepare for testing, a pen marking was made 30 mm from the top and bottom of the specimen, and a discrete minor groove was created at the specimen’s center to induce rupture in the strain gauge fixation, as recommended in [11]. Table 2 presents the key findings from tension experiments for PET-G and ABS. Figure 4 shows the specimens after testing; the prevailing failure mode was brittle, independently of polymer type. Ziemian et al. [48] and Jap et al. [46] pointed out that such a mode for ABS specimens is expected due to the rigid glassy material behavior. From Table 2, the flow stress represents the offset yield point where a plastic strain of 0.5% occurred; this is the standard value for polymers. The tensile strength at break and the elongation were the values where the sample failed. The mechanical characteristics of tensile strength at break and the elongation are very similar to those of a manufacturer (3DLab^®^) considering the PET-G polymer. The same is true for the ABS polymer and, also considering Montero et al.’s [44] results.

**Figure 5:**
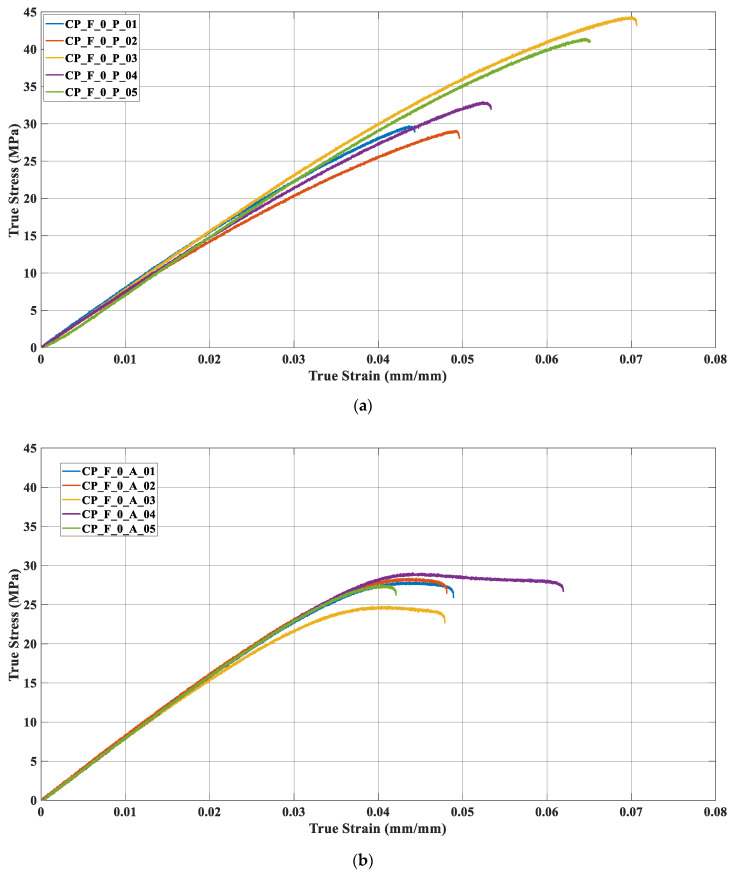
Tensile experiment curves (**a**) PET-G (**b**) ABS.

Figure 5 depicts the true stress versus true strain curves for each of the five samples tested, considering both polymers. Based on the results, it is evident that the samples exhibited comparable behavior, which is why their curves have been so close, see Figure 5a,b. For PET-G samples, samples 3 (yellow) and 5 (green) displayed tensile stresses approximately 15 MPa higher than the remaining three samples, Figure 5a. Similarly, in the case of ABS, samples 3 (yellow) and 4 (purple) deviated from the other samples. Sample 4 presented a more significant strain than the others. Results generally showed that ABS samples were more rigid than PET-G samples.

**Figure 6:**
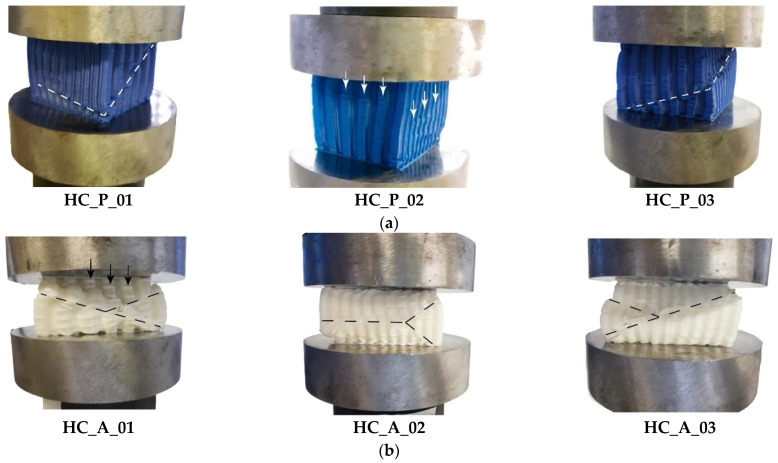
Honeycomb samples after the compression testing: (**a**) specimens in PET-G; (**b**) specimens in ABS.

All honeycomb specimens in both polymers underwent uniform compressive testing. Figure 6 outlines the samples after crushing; the shear stress flow seems to provoke instability in the structure, as discussed by [49,50], except HC_P_02. The figure’s white and black dashed lines highlight the slide plane in the faces. The samples HC_A_01, HC_A_02, and HC_A_03 experienced different characteristic failure modes related to compression testing compared to PET-G specimens. HC_A_01 and HC_A_03 presented shearing along a single plane (in one face) and HC_A_02 “y” shaped failure; those modes were due to glassy material behavior. Samples HC_P_01 and HC_P_03 failed according to a shearing along a single plane but in perpendicular faces, as seen in Figure 6a. The planes intersected at a point where the faces were perpendicular to each other.

**Figure 7:**
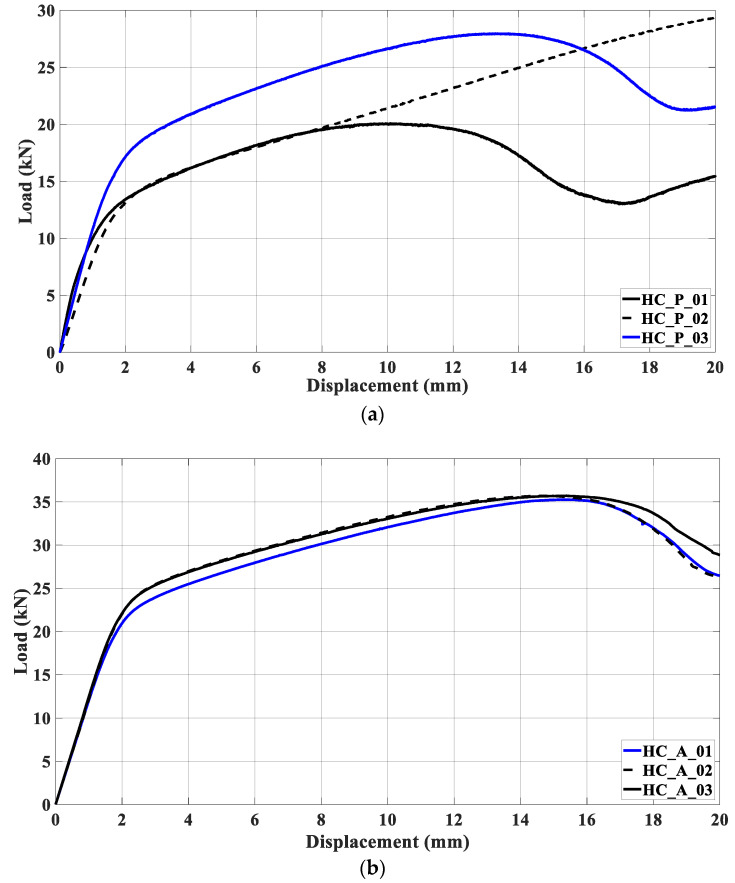
Load versus displacement curves for compression testing concerning (**a**) PET-G and (**b**) ABS polymers.

Figure 7 depicts the load-displacement curves after compression testing. The strain-hardening in ABS samples was more accentuated than that suffered by PET-G specimens. The compression curves of ABS samples are similar, suggesting uniformity in the manufacturing process. Samples HC_P_01, HC_P_03, and HC_A_01 to 03 exhibited a certain amount of strain-softening. Nevertheless, this is more pronounced in PET-G samples due to their lower rigidity. In HC_P_02, Figure 7a, the load-displacement curve is compatible with the failure mode shown in Figure 6a; the compression led to a crushing mode.

The main results obtained from the compression testing are summarized in Table 3. The mechanical properties were determined according to [10]. The ultimate strength refers to the maximum compressive capacity, considering the sample cross-sectional area. The compressive modulus represents the compressive chord modulus applying a specific equation of [10], and the findings evidence the greater ABS rigidity. The difference of both values is about 7%. The standard method involves two-point slope calculations over the linear region of the load-displacement curve. The deflection stress reports a specific value of strain of about 2%.

### 3.3. Experimental Setup

The tensile testing was quasi-static using an Instron 8001 setup with a maximum load capacity of 100 kN and maximum displacement of 100 mm. The compressive testing was also quasi-static with the same experimental setup, a maximum load capacity of 100 kN, and a displacement of 20 mm. The 3D-printed samples were placed between the two circular platens, as shown in Figure 3a,b. The test speed for tension was 1 mm/min slower than recommended for Type 1 specimens according to the standard. However, this speed still resulted in a rupture time between 0.5 and 5 min, which aligns with the test method requirements. 

For compression testing, the printed honeycombs were also placed between the two platens, see Figure 3c. The test speed for compression was 3 mm/min, specified in the standard [10] (item 11.5).

**Figure 8:**
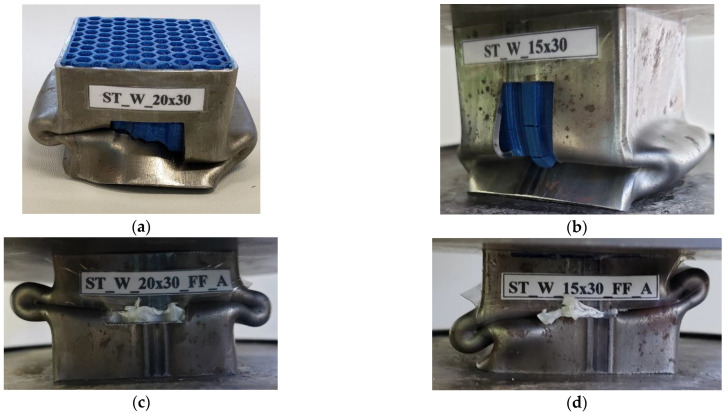
Deformed specimens after axial compression (**a**,**b**) PET-G (**c**) and (**d**) ABS.

The hybrid energy absorbers were tested using an Emic universal testing machine with a loading capacity of 200 kN and a maximum displacement of 20 mm, see Section 4.2. The test speed for compression was 3 mm/min. During axial loading compression, the specimen’s height was reduced by one-third due to compressive displacement. The progress of the crush testing was hindered in filled specimens as the patterned windows closed during compression or the core material came out of the window, see Figure 8. 

As a result, the energy absorbers acted as a solid block, ensuring stable energy absorption, and the sample’s height did not allow for the formation of another lobe. Non-filled samples stopped at the same stroke during testing.

## 4. Results and Discussion

This section discusses the findings regarding the axial loading of the specimens. Table 4 shows the results of the performance parameters, while Table 5 displays the mass of the samples. The performance parameters were obtained using Equations (1)–(6) in Table 1. Section 4.1 presents the results and discussion about non-filled pieces, while Section 4.2 concerns the filled samples. Both appear under the nomenclature detailed in Section 3.1. It is worth noting that all parameters were calculated based on a sample compression displacement of 20 mm for the reasons stated in Section 3.3.

Comparing the performance parameters shown in Table 4, the results lead to the following discussion. The tubes had the same cross-section (50 × 50 mm^2^) and height (60 mm). The windows’ width was 30% or 40% of the cross-section width, and the height was 33% or 50% of the tube height. The windows’ dimensions are presented in Section 3.1. The windows arranged on the faces of energy absorbers allow a change in stiffness measured in the elastic phase until the peak force.

### 4.1. Results of Non-Filled Samples

**Figure 9:**
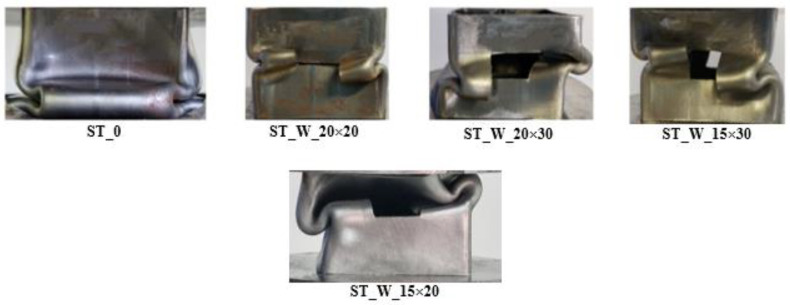
Energy absorber final deformation considering non-filled samples.

Figure 9 depicts all non-filled specimens after loading compression testing. As mentioned in Section 3.1, these specimens were designed to reach symmetric collapse mode. The testing results supported the findings of [30], as in the present paper, the dimensions used for the triggers led to this type of failure.

**Figure 10:**
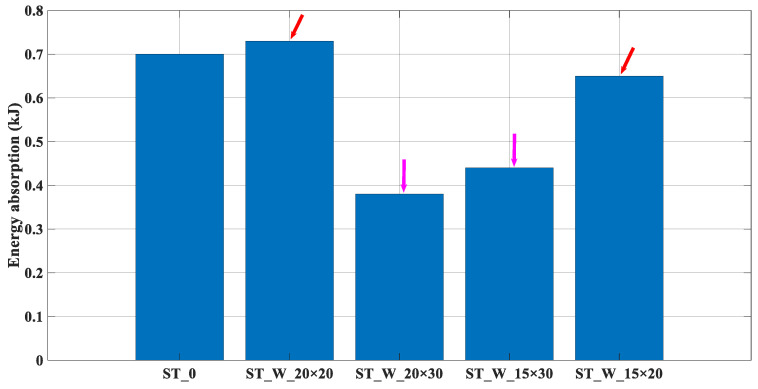
E_a_ (kJ)—Energy absorption (ST_0; ST_W_20 × 20; ST_W_20 × 30; ST_W_15 × 30; ST_W_15 × 20).

From an analysis of the energy absorption, Figure 10, the greater the ratio ‘*b*/*h*’, the more significant the performance parameter, and the red arrows highlight such results.

The samples ‘ST_W_20 × 30’ and ‘ST_W_15 × 30’ have more significant material removal from the energy absorber side face (magenta arrows). The energy absorption for the sample ‘ST_W_20 × 20’ is 4.3% greater than the ST_0 sample, contrary to the results found by [30]. However, the other windowed samples followed the tendency pointed out by [30], except for the percentage values 84.2% (ST_W_20 × 30) and 7.7% (ST_W_15 × 20). 

**Figure 11:**
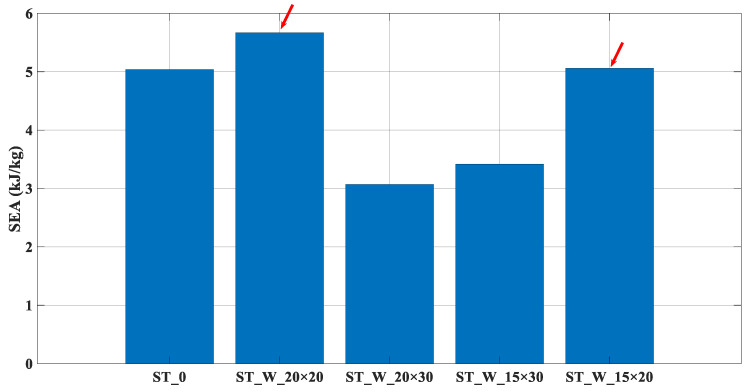
SEA (kJ/kg)—Specific Energy Absorption (ST_0; ST_W_20 × 20; ST_W_20 × 30; ST_W_15 × 30; ST_W_15 × 20).

Figure 11 depicts that the better performance related to the SEA followed the same tendency; the greater the ratio ‘*b*/*h*’, the more significant the performance parameter; red arrows point out such behavior. According to Table 5, these specimens have the lowest mass and the smallest capacity to absorb energy, Table 4.

**Figure 12:**
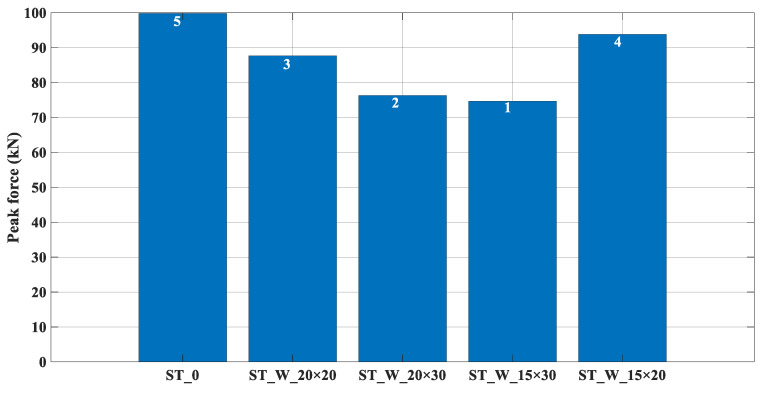
P_peak_ (kN)—Peak force (ST_0; ST_W_20 × 20; ST_W_20 × 30; ST_W_15 × 30; ST_W_15 × 20).

About the peak force, in Figure 12, it is possible to notice that the lower the ratio ‘*b*/*h*’, the lower the peak force (histograms 1 and 2). Also, it is possible to notice that specimen ‘0’ without triggers had the highest peak force (histogram 5). According to [30], windowed samples have reduced this performance parameter, which is confirmed in the present work. The order of increasing loadings is indicated by the numbering on the histogram.

**Figure 13:**
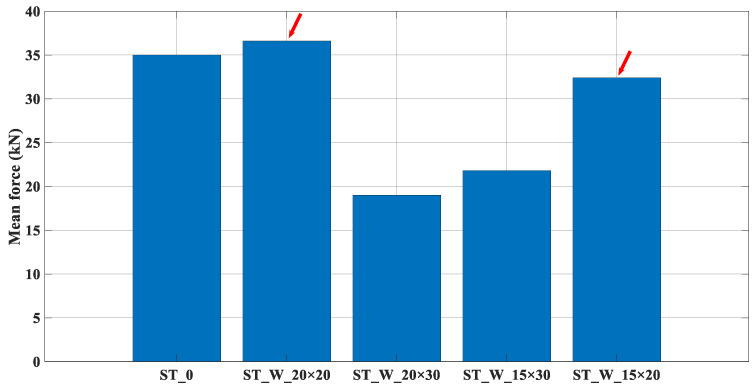
P_m_ (kN)—Mean force (ST_0; ST_W_20 × 20; ST_W_20 × 30; ST_W_15 × 30; ST_W_15 × 20).

Figure 13 shows that the mean force depends on the energy absorption and on the displacement. Its variation among the samples follows the same trend of energy absorption (Figure 10). This parameter measures the efficiency of the energy distribution during compression displacement. The ST_W_20 × 20 and 15 × 20 samples exhibit the highest efficiency in this context, as pointed out by the red arrows.

**Figure 14:**
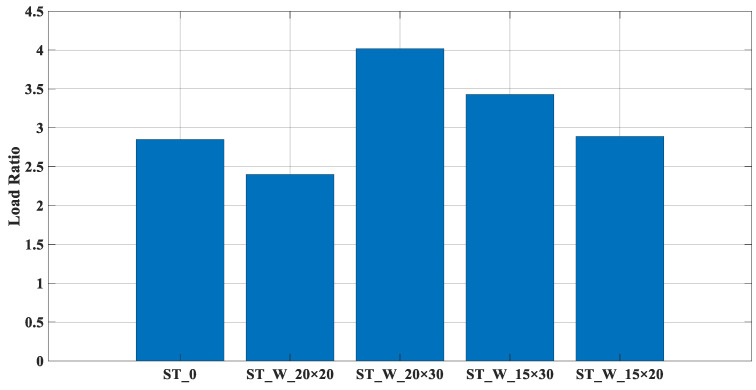
LR (ST_0; ST_W_20 × 20; ST_W_20 × 30; ST_W_15 × 30; ST_W_15 × 20).

In Figure 14, the LR performance parameter is shown. This measures the energy dissipation capacity based on a displacement of 20 mm for all the samples. Among the specimens tested, the ‘ST_W_20 × 30’ and ‘ST_W_15 × 30’ had the lowest performance, indicating a low ratio between peak and mean forces. 

Due to their low energy absorption efficiency, the samples ‘ST_W_20 × 30’ and ‘ST_W_15 × 30’ with the lowest *‘b*/*h’* values had the highest LR parameters. The samples ‘ST_0’ (2.85) and ‘ST_W_15 × 20’ (2.89) had similar values, as seen in Figure 14, but still more than one. The sample ST_W_20 × 20 (2.40) had a better performance despite being greater than one.

**Figure 15:**
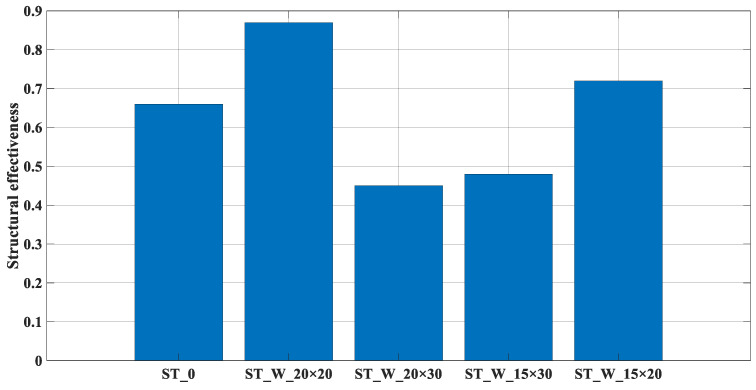
‘*η*’ (ST_0; ST_W_20 × 20; ST_W_20 × 30; ST_W_15 × 30; ST_W_15 × 20).

Figure 15 depicts the parameter ‘*η*’, which compares the mean force to the effectiveness of the material applied to the structure. Notably, the ‘ST_W_20 × 20’ and ‘ST_W_15 × 20’ samples had values near 1, indicating superior performance. Conversely, the ‘ST_W_20 × 30’ and ‘ST_W_15 × 30’ samples exhibited the poorest performance, as highlighted in Figure 14. The evaluation of the ‘0’ sample reveals the evident positive impact of patterned windows on this parameter.

Based on the performance parameter values, the samples ST_W_20 × 20 and ST_W_15 × 20 showed better performance in progressive buckling. The polymeric honeycomb structure was used to fill the samples, and the results are shown in the next Section. The bar graphs repeat the non-filled parameter results to establish a reference for performance comparison.

### 4.2. PET-G and ABS-Filled Samples

The characteristics of the printed honeycomb filling the metal square thin-walled tube were analyzed in Section 3.1, resulting in 77 completed cells in the transverse section measuring 50 × 50 mm^2^. Cellular structures possess an essential feature: their relative density. It is the ratio between the density of the cellular material, represented by ‘ρ*’, and the material density, represented by ‘ρ_s_’, that the cells are made of. 

According to the material supplier, PET-G has a density of 1270 kg/m^3^, while ABS has 1060 kg/m^3^. These materials have a relative density of about 20% and 25%, respectively. According to research by [51], when the relative density reaches around 30%, the cellular structure behaves more like a solid with isolated pores rather than a cellular structure.

The energy absorption decreased in the windowed specimens independent of the polymer type. The decrease in energy absorption comparing the ‘ST_0_FF_P’ with ‘ST_W_20 × 20_FF_P’ was about 13.6% and, for the ABS specimens ‘ST_0_FF_A’ and ‘ST_W_20 × 30_FF_A’, the drop in values was approximately 24.4%. This result did not corroborate with [30], which presented lower decreases in the windowed samples.

**Figure 16:**
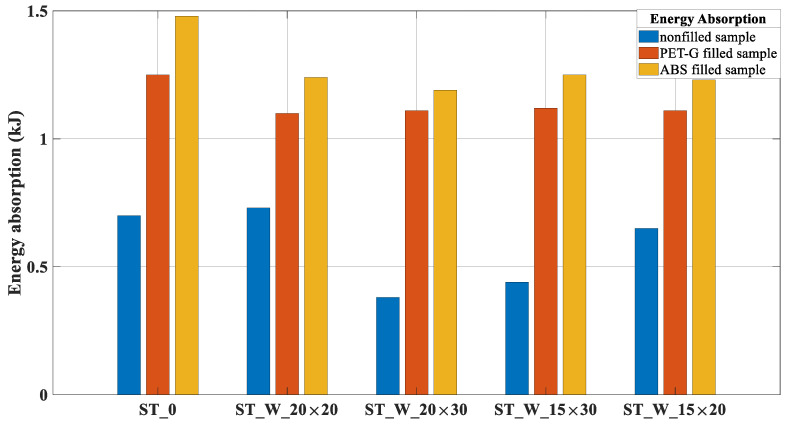
Energy absorption for PET-G/ABS filled samples (ST_0; ST_W_20 × 20; ST_W_20 × 30; ST_W_15 × 30; ST_W_15 × 20) compared with non-filled samples.

In addition, Figure 16 points out that, independent of the polymer, the presence of windows with different sizes did not significantly affect the performance of hybrid tubes. This is most evident in the PET-G samples and less noticeable in the ABS hybrid tubes. Nevertheless, the ABS specimens presented a higher absorption than PET-G samples, considering the same windowed tube. The increase in the energy absorption between the samples ST_0_FF_P and ST_0_FF_A was about 18.4%; 12.7% for samples ST_W_20 × 20_FF_P and ST_W_20 × 20_FF_A; 7.2% for samples ST_W_20 × 30_FF_P and ST_W_20 × 30_FF_A; 11.6% for samples ST_W_15 × 30_FF_P and ST_W_15 × 30_FF_A and 10.8% for samples ST_W_15 × 20_FF_P and ST_W_15 × 20_FF_A.

**Figure 17:**
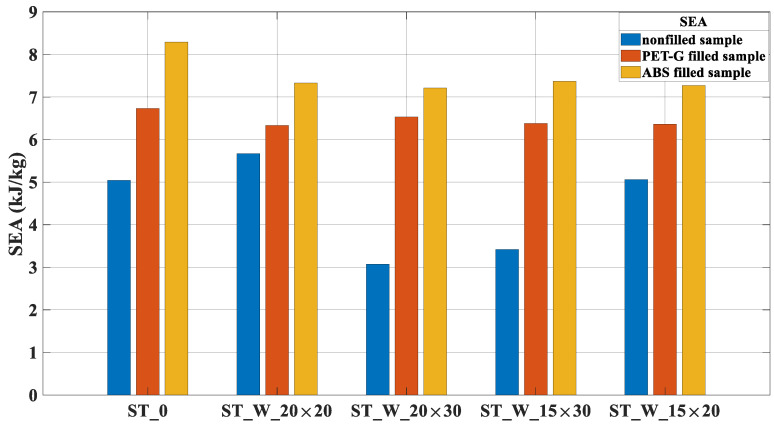
SEA for PET-G/ABS filled samples (ST_0; ST_W_20 × 20; ST_W_20 × 30; ST_W_15 × 30; ST_W_15 × 20) compared with non-filled samples.

The SEA parameter, Figure 17, was more effective for the ABS samples, presenting results higher than those of PET-G samples. Such behavior is justified by the higher energy absorption capacity (Table 4) and lower mass (Table 5). The augmentation between the samples ‘ST_0’ filled with ABS and PET-G was about 23.2%; for the samples, ST_W_20 × 20_FF was 15.8%; for samples, ST_W_20 × 30_FF was 10.4%; ST_W_15 × 30_FF was 15.5% and 14.3% for the 15 × 20 sample.

**Figure 18:**
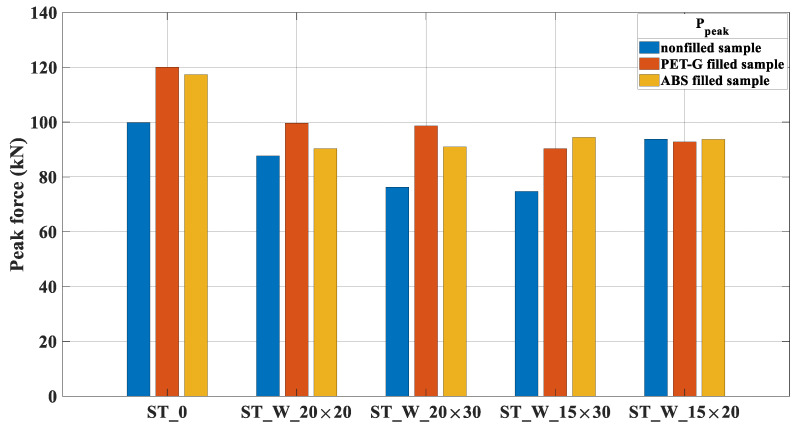
P_peak_ for PET-G/ABS filled samples (ST_0; ST_W_20 × 20; ST_W_20 × 30; ST_W_15 × 30; ST_W_15 × 20) compared with non-filled samples.

The peak force is a parameter used to assess the performance of crash boxes, Figure 18. This force measures the initial peaking load due to the impact of another mass in an axial direction. This is the force needed to cause the first folding. Usually, it is expected to be as low as possible because it determines how much force is necessary to drive the energy absorber to deform before transferring the force effect to the car body.

In some PET-G samples, the peak forces exceed those of ABS polymers, such as ST_0_FF_P, ST_W_20 × 20_FF_P and ST_W_20 × 30_FF_P. The difference between peak forces values for the samples ST_W_20 × 20_FF_P/ST_W_20 × 20_FF_A and ST_W_20 × 30_FF_P/ST_W_20 × 30_FF_A is about 10.3% and 8.4%, respectively. A reasonable explanation for this behavior lies in the less rigid nature of PET-G, which, when deformed further, leads to more significant internal pressure within the thin-walled tube.

The samples ST_W_15 × 30_FF_A and ST_W_15 × 20_FF_A were the exceptions. From these findings, the difference was about 4.54% between the samples ST_W_15 × 30_FF_P and ST_W_15 × 30_FF_A. The sample ST_W_15 × 20 is the one with the smallest trigger area.

As indicated in Figure 18, the filled tubes exhibit significant peak forces regardless of the polymer material used. This observation was noted by [24] and has been corroborated by the present research. The high forces can be attributed to the lateral confinement of the core within the tube, which generates pressure on the tube’s faces, as seen in ST_0_FF_P and ST_0_FF_A. It is worth noting that the ST_W_15 × 20, ST_W_15 × 20_FF_P, and ST_W_15 × 20_A samples have the smallest window area (300 mm^2^), and the peak forces are evenly balanced. Therefore, it could be concluded that the patterned window did not significantly affect the sample’s performance in this case.

**Figure 19:**
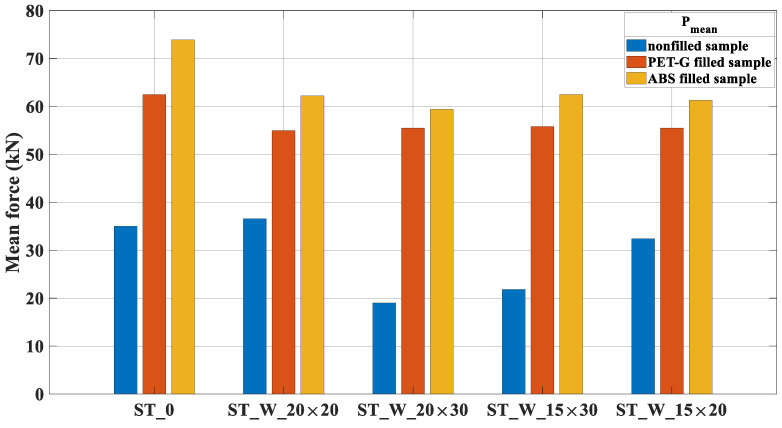
P_m_ for PET-G/ABS filled samples (ST_0; ST_W_20 × 20; ST_W_20 × 30; ST_W_15 × 30; ST_W_15 × 20) compared with non-filled samples.

The mean force, Figure 19, shows the energy absorbed during compression displacement. This accomplishment parameter was better for ABS specimens than those of PET-G. The highest value registered was 13.09% between ST_W_20 × 20_FF_A and ST_W_20 × 20_FF_P.

**Figure 20:**
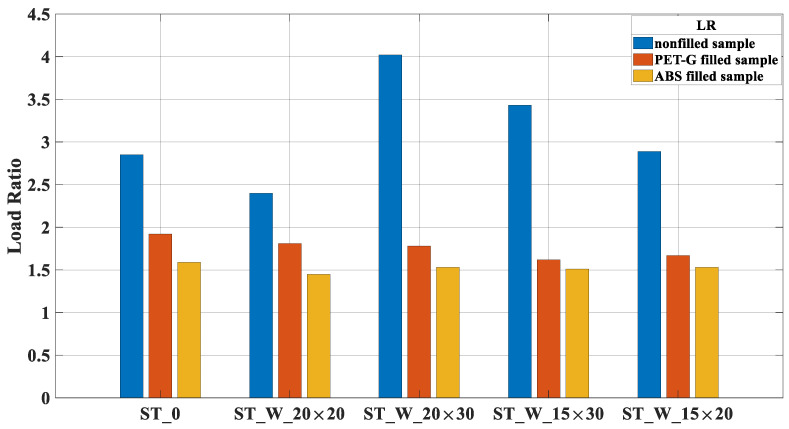
LR for PET-G/ABS filled samples (ST_0; ST_W_20 × 20; ST_W_20 × 30; ST_W_15 × 30; ST_W_15 × 20) compared with non-filled samples.

The LR parameter denotes the ratio between the peak and mean forces, Figure 20, and its value is considered ideal when it is close to one (01). For most samples, the peak forces are more significant in those of the PET-G polymer (Figure 18), while the mean force was consistently more outstanding in the ABS samples, see Figure 19. For the samples in ABS, this equilibrium is better reached, especially for the ST_W_20 × 20_FF_A followed by the ST_W_15 × 30_FF_A and then by the ST_W_20 × 30_FF_A and ST_W_15 × 20_FF_A samples. Comparing the samples filled with PET-G and ABS, the ST_0 samples presented a difference of about 20.75%; sample ST_W_20 × 20_FF was 24.83% and 16.34% for ST_W_20 × 30_FF. The LR significantly improves with the presence of the polymeric core when compared to the outcomes of non-filled samples. Despite a rise in peak forces (Figure 17), there is a noteworthy increase in mean force (Figure 19).

**Figure 21:**
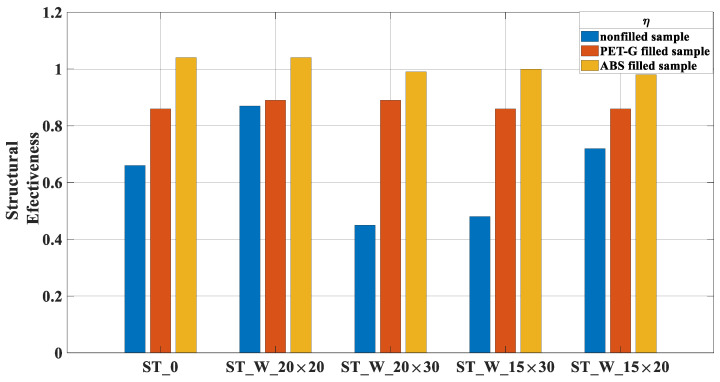
‘*η*’ for PET-G/ABS filled samples (ST_0; ST_W_20 × 20; ST_W_20 × 30; ST_W_15 × 30; ST_W_15 × 20) compared with non-filled samples.

Considering that ‘*η*’, Figure 21, compares the mean force with available strength provided by the material applied to the energy absorbers, the ABS samples had an equilibrium in these values very close to 01 (one), demonstrating that these samples effectively used the strength available in the composite structure. On the contrary, the *η* values were all under 0.9 for the PET-G samples.

**Figure 22:**
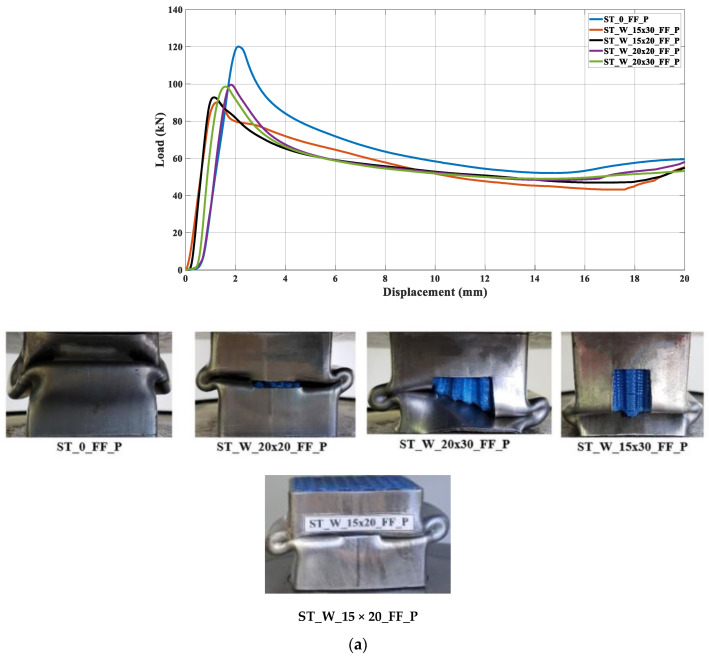

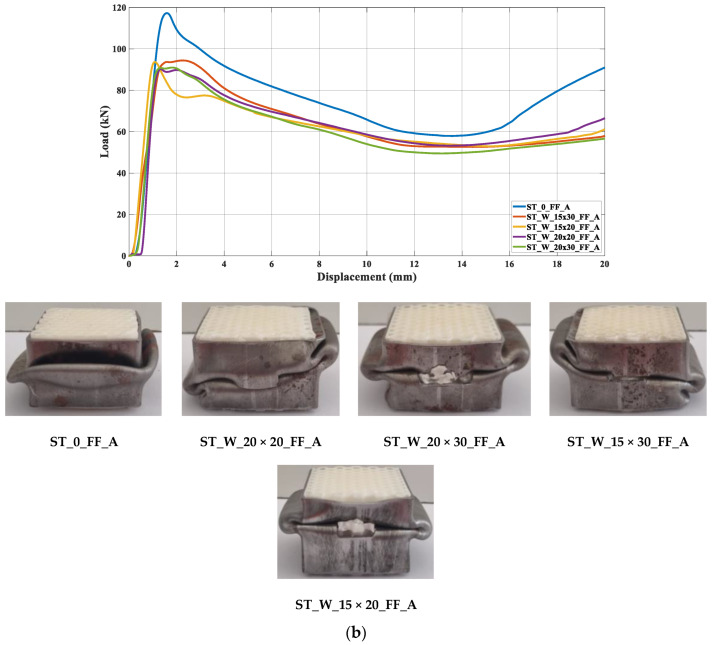
Load curves versus displacement considering the specimens filled with (**a**) PET-G and (**b**) ABS followed by the samples after crushing.

Figure 22a,b illustrates the curves concerning the energy absorbers under axial crushing. A sample steadying in the testing machine led to an offset before the load increased. The filled specimen ‘0’ had the major peak force and energy absorption compared to the other specimens, independent of the core material. Figure 16, Figure 17, Figure 18, Figure 19, Figure 20 and Figure 21 and Table 4 also support this result.

The samples’ behavior was almost identical in the elastic phase before the load peaked. Figure 22a illustrates that the samples’ behavior in the elastic zone is parallel, and the differences among the curves mentioned earlier were due to loading accommodation. The elastic phase for specimens ST_W_15 × 30_FF_P and ST_W_15 × 20_FF_P matched, with a slight deviation for the 20 × 30 mm^2^ sample. A greater offset was detected in specimen ‘0’ and 20 × 20 at the elastic region, even though both specimens start their deformation at zero. However, specimens ST_0_FF_P and ST_W_20 × 20_FF_P showed correspondence in the elastic zone.

Based on Figure 22b, the ABS hybrid specimens show a more uniform elastic phase than the PET-G specimens. This consistency suggests that the windows have not significantly impacted the slope of the curve at this phase. Additionally, using ABS material in the core may ensure equitable behavior. Figure 18 exhibits that the peak forces have values near each other. After the elastic phase, the effects of the polymer window and core become more apparent in Figure 22a,b.

The stabilization in the testing machine was more extensive in sample ‘0’ than in the windowed samples, evidencing the positive effect of the triggering mechanism at the beginning of the crushing process. The offset between the curves in the elastic phase could not be attributed only to the material type used in the core because, if so, the windowed sample curves would be further apart in this phase. Thus, it may be inferred that the presence of the windows was a major factor, especially after the peak force. As said, the patterned windows trigger the specimens, inducing the energy absorber’s metallic part to primary deformation, predominating in the elastic phase over the core. Such a configuration was able to be observed because the core was not bonded to the metallic matrix.

**Figure 23:**
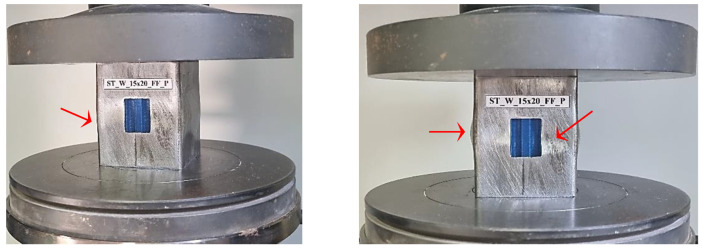
Specimen ST_W_15 × 20_FF_P at the beginning of the deformation process.

**Figure 24:**
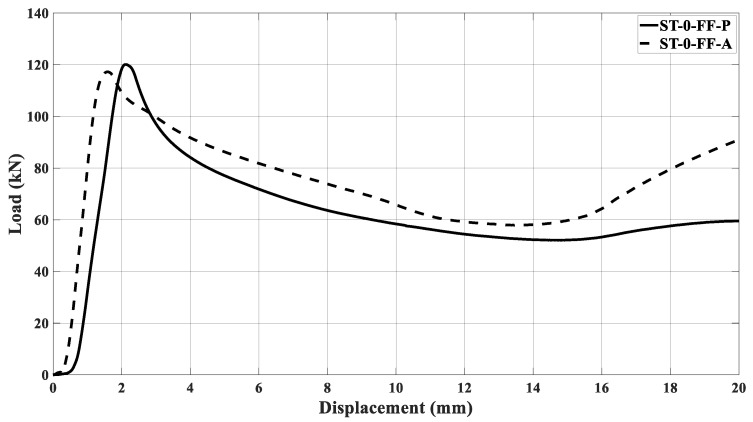
Load curves versus displacement for ST_0 specimen considering being filled with PET-G and ABS.

**Figure 25:**
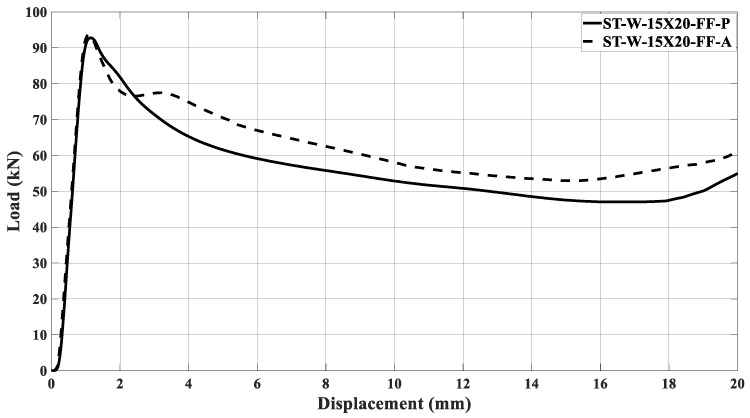
Load curves versus displacement for ST_W_15 × 20 specimen considering being filled with PET-G and ABS.

**Figure 26:**
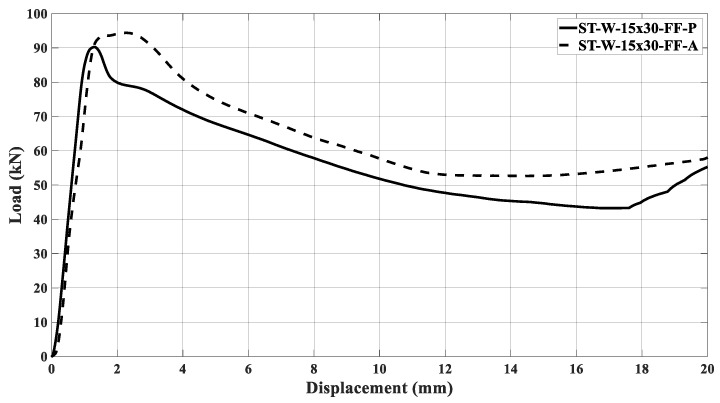
Load curves versus displacement for ST_W_15 × 30 specimen considering being filled with PET-G and ABS.

**Figure 27:**
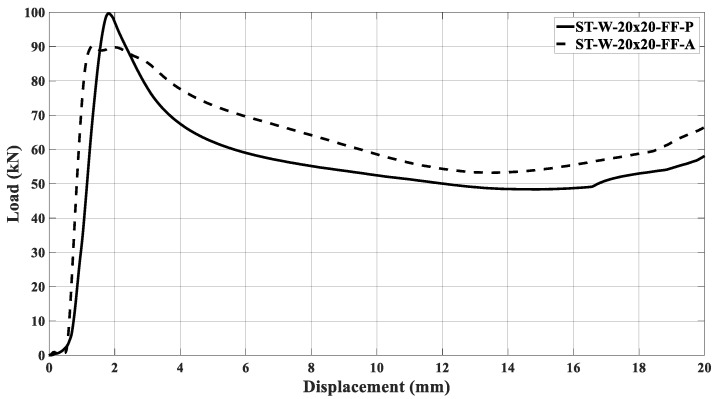
Load curves versus displacement for ST_W_20 × 20 specimen considering it filled with PET-G and ABS.

**Figure 28:**
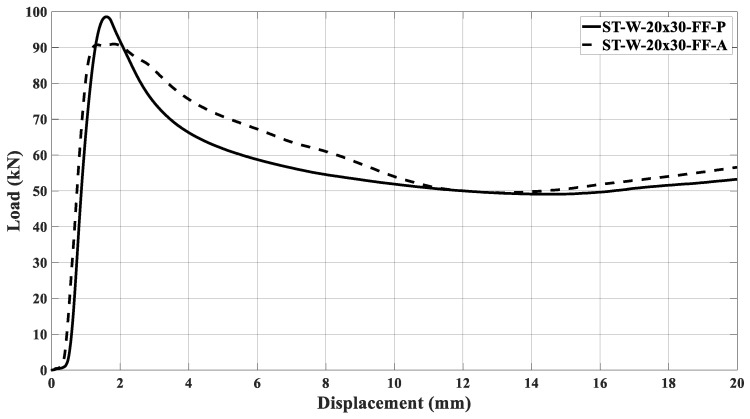
Load curves versus displacement for ST_W_20 × 30 specimen considering it filled with PET-G and ABS.

Figure 23 illustrates the ST_W_15 × 20_FF_P initial deformation, highlighted by the red arrows. The same deformation mechanism was noticed for all specimens where the steel stood out during the crushing load. The slope in the elastic zone for sample ST_W_15 × 20_FF was about 131 kN/m; 119 kN/m for ST_W_15 × 30_FF; 98 kN/m for ST_W_20 × 30_FF, independent of the core material, showing that an increase in the window area leads to lower stiffness which corroborates the above statement. Considering the ST_W_20 × 20_FF_P, the stiffness was about 126 kN/m.The composite steel/polymer undoubtedly withstood more loading than the metallic samples, as discussed above, see Table 4. Figure 24, Figure 25, Figure 26, Figure 27 and Figure 28 depict the curves load *versus* displacement in pairs considering the same energy absorber windowed geometry, highlighting the contribution of different core polymer materials. 

In the plastic phase, after the peak load, the core contribution in the crushing process became more noticeable, even pointing out that the ABS core led the absorbers to better performance, Figure 24, Figure 25, Figure 26, Figure 27 and Figure 28. The slope in the elastic phase was 96 MPa for PET-G and 158 Mpa for ABS in specimen ‘0’, demonstrating greater stiffness for the ABS specimen, as verified in the work of [52].

**Figure 29:**
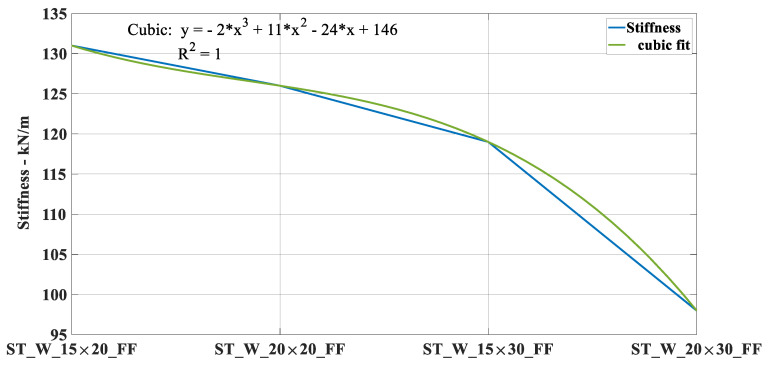
Curve stiffness versus energy absorption considering cubic basic fitting.

Figure 29 depicts the stiffness of the filled samples, and from Figure 24, Figure 25, Figure 26, Figure 27 and Figure 28, the slope was considered the same for both specimens with PET-G and ABS cores. A basic fitting applying a cubic fit considering the stiffness values in the elastic phase presented an R^2^ regression factor equal to one (01). For that, the stiffness values in descending order were considered, i.e., ST_W_15 × 20_FF, the highest value, and the lowest value ST_W_20 × 30_FF. This highlights that as the window area increases, the stiffness in the elastic phase decreases, such as a cubic curve.

In addition, using an interaction evaluation between two categorical factors, it is possible to evaluate how one factor depends on the value of the second one. Minitab Statistical Software^®^ (version 17) allowed such analysis considering the following groups where the relation b/h was the common factor in all analyses. The plots displayed the mean for the levels of one factor, in case $E_{a}$, $P_{m}$, $P_{peak}$, *η* and *LR* versus b/h considering non-filled and PET-G/ABS-filled energy absorbers.

**Figure 30:**
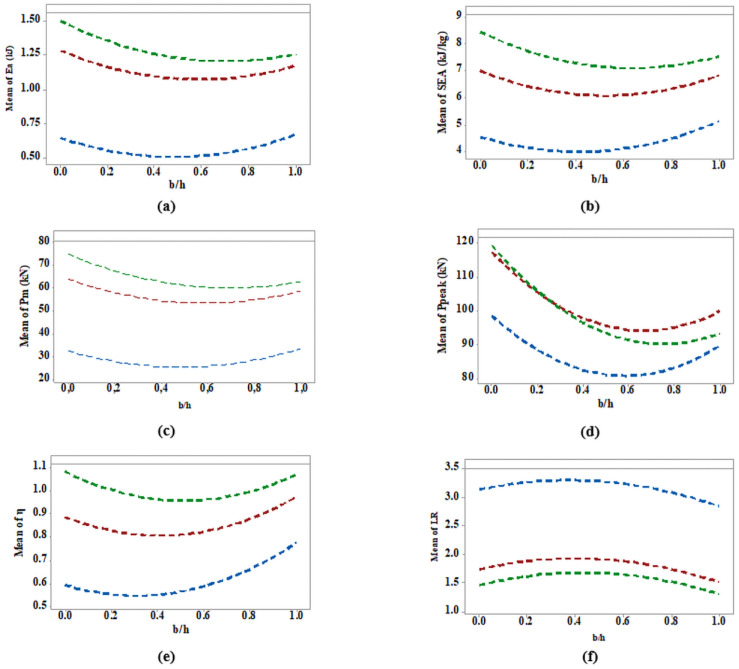
Interaction plots for performance factor—**blue** curve for unfilled energy absorbers; **red** curve PET-G filled energy absorbers; **green** curve ABS filled energy absorbers. (**a**) $E_{a}$ versus b/h; (**b**) *SEA* versus b/h; (**c**) $P_{m}$ versus b/h; (**d**) $P_{peak}$ versus b/h; (**e**) *η* versus b/h; (**f**) *LR* versus b/h.

The graphics in Figure 30 show no significant interaction between the factors, regardless of whether the energy absorbers were filled or the material used. Also, the continuous response curves were not linear but parabolic. ABS energy absorbers (green curves) were in better condition for specific performance parameters, such as $E_{a}$, *SEA*, $P_{m}$, *η* and *LR*, confirming some previous analyses, Figure 16, Figure 17, Figure 18, Figure 19, Figure 20 and Figure 21. In Figure 30d, there was an exception where the $P_{peak}$ values for samples filled with polymeric honeycomb intersected at b/h values between 0.2 and 0.3. This interaction effect implies that the correlation between $P_{peak}$ and b/h is somewhat influenced by the material used in the core. $P_{peak}$ was marginally inferior for energy absorbers filled with ABS when b/h was above 0.3 and slightly superior under this value.

Upon conducting additional analyses through an optimization response, it was evident that by optimizing the performance indicator, $P_{peak}$, significantly impacted the composite desirability parameter (output from the optimization analysis). This finding confirms the correlation between the $P_{peak}$ value and the mechanical design of the sample, as discussed later.

It should be noted that assessing the performance of energy absorbers, especially those with a polymer core, can be a challenging task based exclusively on interpreting the force x displacement curves or the interaction plots analysis considering the various performance indicators with different readings (Table 4). A response optimizer tool that shows how different experimental settings affect the predicted responses for a stored model can help pinpoint the most optimized configuration. It is useful when evaluating the impact of multiple variables on a response, helping to identify the best b/h ratio and core material from the testing samples carried out.

Table 6 shows each response’s requirements (settings) to achieve such a goal, asking to minimize, target, maximize, or not optimize the response for the performance indicators ($E_{a}$, SEA, $P_{peak}$, $P_{m}$, LR and *η*). The responses concerning the $E_{a}$, SEA, LR, and *η* were kept the same in all trials, in case maximized response for $E_{a}$ and SEA and target in 01 (one) for LR and *η*. 

In addition, the option ‘Importance’, which determines the relative status of multiple response variables, was first kept at 01 (one), the default value, for all performance indicators. It can vary from 0.1 to 10. Four experiments were carried out where the fourth was the same as Exp. 2 (Table 6), but the ‘Importance’ option was chosen to be 10 for the $E_{a}$, SEA, and $P_{peak}$.

Other variables considered in the response optimization were the b/h ratio, which considered the width and height, respectively, of the window in the windowed samples that could be filled with the honeycomb core or not. Restating the ratios ‘1’ for samples window 20 × 20, 0.75 for samples window 15 × 20, 0.67 for samples window 20 × 30, and 0.5 for samples window 15 × 30. The core presence was treated considering ‘0’ for non-filled samples, ‘1’ for samples filled with PET-G, and ‘2’ for those filled with ABS.

Individual (*d*) and composite (*D*) desirability assess how well a combination of variables satisfies the goals defined for responses. Individual desirability evaluates how the settings optimize a set of responses overall.

Evaluating experiment 01 (Exp. 01—Table 6), ‘*D*’ was about 80%. Individual desirability was more effective in maximizing the $E_{a}$ (*d* = 75.5%) and SEA (*d* = 79.6%) than minimizing the $P_{peak}$ (*d* = 65.6%). The response values for b/h and the presence or absence of the polymer core were 0.82 and ABS (2), respectively. 

For experiment 2 (Exp. 02—Table 6), ‘*D*’ was about 79.3%. The individual desirability denoted that maximizing $E_{a}$ (*d* = 75.6%), SEA (*d* = 79.6%), and $P_{m}$ (d = 75.7%) led to a ‘*D*’ very close to that of the Exp. 01. $P_{peak}$ was minimized, reaching a *d* = 65.6%. The experiment showed that maximizing the $P_{m}$ did not favor the composite desirability. The response values for b/h and the presence or absence of the polymer core remained the same as in experiment 01.

The third experiment (Exp. 03) maximized the $E_{a}$ (*d* = 97.0%), SEA (*d* = 92.4%), and $P_{m}$ (*d* = 97.1%). $P_{peak}$ was not optimized, resulting in a composite desirability, ‘*D*’, of about 94%. The response values for b/h and the presence or absence of the polymer core were 0 (no window) and an undefined material when the value was 1.52, respectively. Notice the individual desirability for $E_{a}$, SEA and $P_{m}$ in Exp. 03 was better than the other experiments, showing that $P_{peak}$ exerted an essential role in the analysis.

In addition, when the material in Exp. 03 is constrained to be equal to 1 (PET-G) or 2 (ABS) values, the composite desirability reaches the values *D* = 78.7% or *D* = 0, respectively. Compared to other experiments, the ‘*D*’ value for Pet-G is lower, and for ABS, such a condition could not be accomplished using samples without windows. Therefore, the mechanical behavior of windowed samples seems more consistent, resulting in a more efficient optimization of composite desirability (*D*). In this experiment, the optimal response showed improvement compared to the previous two attempts. However, it suggests that the optimization was targeted toward a non-existent material.

The fourth experiment (Exp. 04) applied the same settings of Exp. 02, but the option ‘Importance’ setting was fixed as ten (10) for the performance indicators $E_{a}$, SEA, and $P_{peak}$. The composite desirability was 74.4%, and the individual desirability was $E_{a}$ (*d* = 75.6%), SEA (*d* = 79.6%) and $P_{m}$ (*d* = 75.7%), and $P_{peak}$ (65.6%). These results did not indicate any impact on the optimization process when setting the ‘Importance’ to 10. The composite desirability is the same as Exp. 02.

Table 7 depicts the optimized values ‘y’ for each performance indicator to be expected if the current variables (b/h and core material) are adopted in each experiment. 

Following the b/h ratio of experiment 2 in Table 7, an optimized patterned energy absorber was proposed, denominated ST_W_16.4 × 20_FF_A. The samples are stiffer with a height of 20 mm, as depicted in Figure 29; hence, the size was maintained at 20 mm while the width was adjusted, leading to a 9.3% larger area than sample ST_W_15 × 20_FF. As expected, the new sample had a mean stiffness of 126.6 kN/m between ST_W_15 × 20_FF and ST_W_20 × 20_FF.

**Figure 31:**
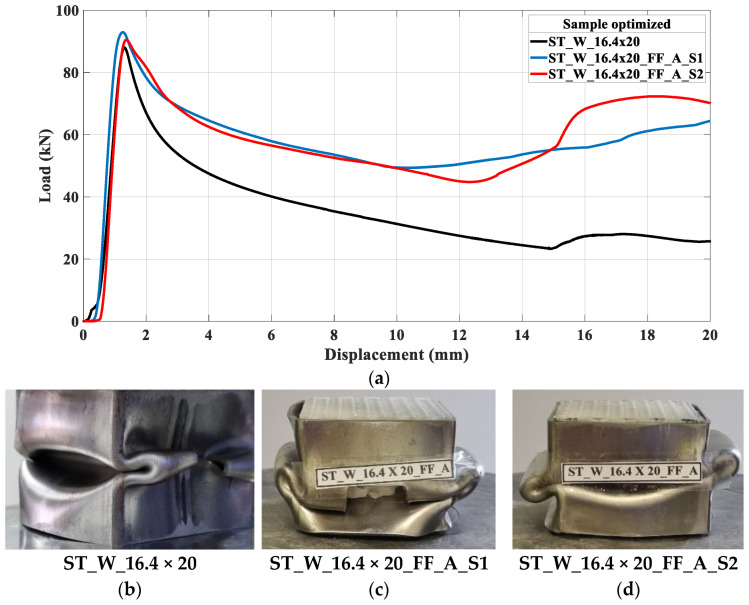
(**a**) Curves force versus displacement of the optimized samples; (**b**) non-filled sample; (**c**) filled sample ‘S1’; (**d**) filled sample ‘S2’.

Figure 31 illustrates the curves of crush testing on a non-filled sample and two filled pieces. The three specimens presented a symmetric collapse mode, as seen in Figure 31b–d. The mass values were 128.1 g for the non-filled specimen, 164.9 g for filled sample one, and 164.2 g for sample two. As observed in other samples, the elastic phase was too close to each other, specifically for the filled samples. The peak force for filled specimens was greater than for non-filled samples, and the reason for this was presented previously. The curves for filled samples remained coincident until a displacement of 10 mm, after which they started to diverge. Table 8 shows the performance parameter, Table 1, calculated for experimental curves (ST_W_16.4 × 20_FF_A_S1 and ST_W_16.4 × 20_FF_A_S2) and compared with the optimized results, Table 7—Exp. 02.

The fifth column of Table 8 indicates that the most significant discrepancy occurred between experimental and optimized values referent to LR and *η* parameters. However, the percentage is still small.

## 5. Conclusions

Honeycomb structures have been extensively studied in scientific literature. Additionally, polymer materials are widely applied in various technological applications, such as creating cellular structures using the FDM^®^ technique for 3D printing structures, as outlined in Section 1. In this context, the use of cellular structures as a core material for square steel thin-walled energy absorbers presents a new and innovative approach to engineering solutions. Furthermore, the metallic tubes are windowed at half height. The findings indicate that these openings, along with the polymer core, positively affect crashworthiness by increasing energy absorption capacity, improving the load ratio (LR), and enhancing structural effectiveness (*η*).

The novel hybrid energy absorber reached the following requirements: (1) the ability to increase energy absorption was detected by the performance parameters, especially for the ABS samples; (2) some researchers have pointed out that incorporating a core into thin-walled energy absorbers can increase their energy absorption capabilities, as mentioned in Section 1. Thin-walled metallic tubes filled with the 3D-printed hexagonal honeycomb of polymeric thermoplastic material may also be an upcoming trend. During the present research, we verified that once the printing parameters were well established, the manufacturing process of honeycomb structures was not difficult, and the sample cost was not high; (3) we observed that patterned windows caused most of the samples to fail symmetrically, which is a positive form of failure as it led to more uniform and efficient energy dissipation. Considering the ratio width/height for filled samples as 1.0 (samples 20 × 20), 0.75 (samples 15 × 20), 0.67 (samples 20 × 30), 0.5 (samples 15 × 30), it is possible to notice that the elastic stiffness decreases, according to a third polynomial equation. This means that shorter pieces were more rigid. The optimized sample had a stiffness between the samples ST_W_15 × 20_FF and ST_W_20 × 20_FF. It can be inferred that, when dynamically loaded, windowed specimens can reduce peak force magnitude due to the escape area, lowering vehicle occupants’ risk of neck and head injuries.

An interaction evaluation investigated the relationship between the ratio b/h and the mean values of $E_{a}$, *SEA*, $P_{m}$, *η* and *LR*, depending on whether the energy absorber is non-filled, PETG-filled, or ABS-filled. The findings demonstrated no significant interaction, regardless of energy absorber fill or material. Continuous response curves were parabolic, not linear, but parallel lines indicated no interaction exception to the $P_{peak}$. Such a condition means that each group of energy absorbers can be independently evaluated, improving performance as scenarios change, as outlined in Figure 30.

Choosing the most suitable sample for filled pieces can be challenging since the optimal value for each parameter may not be present in the same specimen. Hence, an optimization approach is the most effective way to suggest a sample with the best performance parameters based on the experimental results (Table 4). The optimization study identified the optimal ratio between b/h (0.82) and the ideal core material. Therefore, for future work, the ideal material will be ABS, and the window geometry will be 16.4 × 20 mm^2^. 

## Figures and Tables

**Table 1:** Summary of performance parameters applied to energy absorbers.

Performance Parameters		Some Considerations
Energy absorption (kJ)		Area under the curve $Force\;\left(kN\right)\;x\;Displacement\;(mm)$.
$E_{a}=\int_{0}^{D}F\left(x\right)dx$	(1)	
Specific Energy absorption (kJ/kg)		It considers the absorbed energy related to reducing the mass of the energy absorber, resulting in improved performance.
$SEA=\frac{E_{a}}{m}$	(2)	
Peak force (kN)		The force is reached at the end of the elastic phase when the absorber starts to undergo strain softening due to the formation of the characteristic wrinkle in dynamic, progressive buckling.
$P_{peak}$	(3)	
Mean force (kN)		*D* is the maximum displacement of the energy absorber.
$P_{m}=\frac{E_{a}}{D}$	(4)	
Load ratio (dimensionless)		It deals with an equilibrium between two characteristic forces of the energy absorbers crush testing. Ideally, the peak force should approach the average force of the system.
$LR=\frac{P_{peak}}{P_{m}}$	(5)	
Structural effectiveness (dimensionless)		This ratio allows for verifying the effectiveness of the material applied through the product area/material characteristic stress and the mean force of the system. ‘*A*’ is the cross-sectional area of each material. *σ*_0_ is the yield stress for each material.
$\eta =\frac{P_{m}}{{\left(A\sigma_{0}\right)}_{steel}+{\left(A\sigma_{0}\right)}_{composite}}$	(6)	

**Table 2:** Mechanical properties in tension experiments for the specimens.

Mechanical Properties	Property Values	
	PET-G	ABS
Flow stress (Mpa)	29.8 ± 4.4	26.7 ± 1.8
Tensile strength at break (Mpa)	30.8 ± 6.2	24.3 ± 1.5
Modulus of elasticity (Mpa)	706.6 ± 27.4	760.6 ± 24.3
Elongation (%)	6.1 ± 1.2	5.4 ± 0.8

**Table 3:** Mechanical properties in compression experiments for the specimens.

Mechanical Properties	Property Values	
	PET-G (MPa)	ABS (MPa)
Ultimate strength	(9.6 ± 1.9)	(13.2 ± 0.1)
Deflection stress	(4.1 ± 0.5)	(5.4 ± 0.1)
Compressive modulus	(252.0 ± 7.8)	(269.8 ± 4.7)

**Table 4:** Results of the steel tubes non-filled and filled with PET-G and ABS.

Energy Absorbers	E_a_ (kJ)	SEA (kJ/kg)	P_peak_ (kN)	P_m_ (kN)	LR	*η*
ST_0	0.70	5.04	99.8	35.0	2.85	0.66
ST_W_20 × 20 (b/h = 1–400 mm^2^)	0.73	5.67	87.7	36.6	2.40	0.87
ST_W_20 × 30 (b/h = 0.67–600 mm^2^)	0.38	3.07	76.3	19.0	4.02	0.45
ST_W_15 × 30 (b/h = 0.5–450 mm^2^)	0.44	3.42	74.7	21.8	3.43	0.48
ST_W_15 × 20 (b/h = 0.75–300 mm^2^)	0.65	5.06	93.8	32.4	2.89	0.72
ST_0_FF_P	1.25	6.73	120.1	62.5	1.92	0.86
ST_W_20 × 20_FF_P	1.10	6.33	99.6	55.0	1.81	0.89
ST_W_20 × 30_FF_P	1.11	6.53	98.6	55.5	1.78	0.89
ST_W_15 × 30_FF_P	1.12	6.38	90.3	55.8	1.62	0.86
ST_W_15 × 20_FF_P	1.11	6.36	92.8	55.5	1.67	0.86
ST_0_FF_A	1.48	8.29	117.3	73.9	1.59	1.04
ST_W_20 × 20_FF_A	1.24	7.33	90.3	62.2	1.45	1.04
ST_W_20 × 30_FF_A	1.19	7.21	91.0	59.4	1.53	0.99
ST_W_15 × 30_FF_A	1.25	7.37	94.4	62.5	1.51	1.00
ST_W_15 × 20_FF_A	1.23	7.27	93.7	61.3	1.53	0.98

**Table 5:** Specimen mass.

Energy Absorbers	Mass (kg)
ST_0	0.13910
ST_W_20 × 20	0.12916
ST_W_20 × 30	0.12354
ST_W_15 × 30	0.12740
ST_W_15 × 20	0.12938
ST_0_FF_P	0.18570
ST_W_20 × 20_FF_P	0.17390
ST_W_20 × 30_FF_P	0.16990
ST_W_15 × 30_FF_P	0.17480
ST_W_15 × 20_FF_P	0.17470
ST_0_FF_A	0.17849
ST_W_20 × 20_FF_A	0.16970
ST_W_20 × 30_FF_A	0.16486
ST_W_15 × 30_FF_A	0.16958
ST_W_15 × 20_FF_A	0.16850

**Table 6:** Settings adopted for optimization experiments.

Performance Indicators	Exp. 01					Exp.02					Exp. 03				
	NO ^1^	Max ^1^	Targ ^1^	Min ^1^	I	NO	Max	Targ	Min	I	NO	Max	Targ	Min	I
$E_{a}$		x			1		x			1		x			1
SEA		x			1		x			1		x			1
$P_{peak}$				x	1				x	1	x				1
$P_{m}$	x				1		x			1		x			1
LR			1		1			1		1			1		1
*η*			1		1			1		1			1		1

^1^ NO: not optimized; Max: maximize value; Min: minimize value; Targ: fixe a desirable value; I: importance value.

**Table 7:** Predict ‘*y*’ values and current variable settings for the optimization experiments.

**Performance Indicator**	**Predict ‘y’ Values after Optimization**		
	**Exp. 01**	**Exp.02**	**Exp. 03**
$E_{a}$	1.2 kJ	1.2 kJ	1.3 kJ
SEA	7.2 kJ/kg	7.2 kJ/kg	7.0 kJ/kg
$P_{peak}$	90.3 kN	90.3 kN	NO
$P_{m}$	NO	60.6 kN	64.0 kN
LR	1.50	1.50	1.45
*η*	1.0	1.0	1.0
**Current variable settings**	**Exp. 01**	**Exp.02**	**Exp. 03**
b/h	0.82	0.82	0 (no window)
Core material	2 (ABS)	2 (ABS)	1.53 (Undefined material)

NO: not optimized.

**Table 8:** Comparison between experimental and optimized results for performance parameters.

Performance Parameters	Sample 01	Sample 02	Mean Value	Difference in %
$E_{a}$ (kJ)	1.18	1.21	1.20	0
SEA (kJ/kg)	7.17	7.37	7.30	1.4% (+)
$P_{m}$ (kN)	57.35	58.09	57.72	5.0% (-)
$P_{peak}$ (kN)	92.96	90.45	91.71	1.6% (+)
*LR*	1.62	1.56	1.59	6% (+)
*η*	0.93	0.94	0.94	6% (-)

+ percentage above the optimization value; - percentage under the optimization value.

## Data Availability

The data that support the findings of this study are available from the corresponding author upon reasonable request.
